# How Does Servant Leadership Foster Employees’ Voluntary Green Behavior? A Sequential Mediation Model

**DOI:** 10.3390/ijerph17051792

**Published:** 2020-03-10

**Authors:** Ma Ying, Naveed Ahmad Faraz, Fawad Ahmed, Ali Raza

**Affiliations:** 1 School of Management, Wuhan University of Technology, Wuhan 430070, China; mying@whut.edu.cn (M.Y.); fawadahmed1@live.com (F.A.); 2 KUBEAC Department, University of Management and Technology, Sialkot 51040, Pakistan; aliraza@skt.umt.edu.pk

**Keywords:** servant leadership, employees’ voluntary green behavior, psychological empowerment, autonomous motivation for the environment, sequential mediation

## Abstract

Employees’ voluntary green behavior (EVGB) is indispensable in realizing organizations’ environmental sustainability objectives. Leaders can act as catalysts to shape the behavior of their employees. On EVGB, noticeably the missing link is investigating the influence of servant leadership and the mechanism through which it operates. Building upon self-determination and psychological empowerment theories, this research examined the impact of servant leadership on EVGB through the simple and sequential mediation of psychological empowerment and autonomous motivation for the environment (AME). Through systematic sampling, dyadic data were collected from 315 pairs of subordinates and supervisors working in the power sector organizations of Pakistan. Results were obtained by employing the partial least squares structural modeling (PLS-SEM) technique with Smart-PLS 3.2.8 software. Findings revealed that psychological empowerment and AME simply and sequentially mediate the influence of servant leadership on EVGB. Implications for theory and organizational practitioners are offered, accompanied by suggestions for future research.

## 1. Introduction

Physical and psychological well-being, together with the collective future of humanity, is dependent on preserving the environment. Environmental issues pose a great challenge for organizations and require the mobilization of considerable resources and competencies to achieve a transition towards sustainability [1]. Corporate greening is a paramount challenge that the organizational word is facing today [1,2] and employees’ play a vital role in overcoming these challenges [3]. Employees’ inertia pertaining to the environmental issues has been a major concern for managers that has led them to explore a wide range of research avenues in the hope of gaining a better understanding of the factors related to environmentally responsible behaviors. In recent years, there has been a burgeoning scholarly interest in examining employees’ extra role behaviors towards the environment [4] and this has offered valuable insights that could shape employees’ green behavior in the workplace. Despite progress on academic and business fronts, organizations continue to face substantial human resource challenges in realizing environmental sustainability initiatives [5,6,7,8]. Ones and Dilchert [9] highlighted that “What organizations do is a function of decisions, behaviors, and performance of their members. Organizational initiatives stem from employees. Therefore, understanding, promoting, influencing, and changing environmental behaviors of employees are keys to environmental sustainability of organizations”. Norton et al. [10] defined employees’ voluntary green behavior that involves personal initiative exceeding organizational expectations, including prioritizing environmental interests, initiating environmental programs and policies, lobbying and activism, and encouraging others. Nurturing employees’ voluntary green behavior not only serves corporate greening objectives, but can also prevent further environmental degradation by positively affecting environmental change.

Leadership is considered to be at the heart of nurturing individual, group and organizational outcomes [11]. Leaders can influence a wide range of diversified organizational outcomes, including environmental considerations [12]. However, the literature has reported the influence of different leadership styles like transformational leadership [13,14], transactional leadership [15], ethical leadership [16], spiritual leadership [17], and responsible leadership [18] on employees’ extra role behaviors towards the environment. Noticeably, the missing link from the researchers’ attention has been the effect of servant leadership and the mechanism through which it operates to influence employees’ voluntary green behavior (EVGB). While comparing servant leadership with other emerging forms of positive leadership, Hoch et al. [19], in their series of meta-analysis, asserted that servant leadership showed its distinctiveness and ability to better explain the variety of outcomes over and above the other forms of leadership. Researchers have reasoned that servant leaders go beyond other types of leaders, primarily in two spheres: focusing on the needs of their followers, and recognizing their own social responsibility [20]. The essence of servant leadership stands on the principle that it should develop their followers, in such a way that they would themselves emerge as servant leaders [21]. Servant leaders consider it their moral responsibility to safeguard the good of all the stakeholders, including employees, customers, and community [22], and environment is no exception to this. The characteristics of servant leadership, such as stewardship, creating value for the community, servanthood, and altruistic calling are implied in the concept of servant leadership [23]. These characteristics highlight that servant leaders act selflessly and are likely to create a sense among their subordinates of caring for the good of wider society. SLs pay too much attention to community service, while EVGB are closely aligned with safeguarding the community through environmental concern. Hence, investigating how servant leadership influences EVGB is genuinely meaningful.

Besides examining the direct association of servant leadership with EVGB, a comprehensive understanding of the mechanisms through which servant leadership leads to such behavior is also under consideration in this research. Scholars maintain that psychological empowerment could potentially be the underlying mechanism, which may describe the relationship of servant leadership with employees’ behavioral outcomes [21,24]. When employees realize that their leadership believes in creating value for society and stands up for their initiatives to protect and care for the environment, they are likely to feel a sense of meaning, competence, self-determination and impact [25]. The concept of empowerment is present in almost all the definitions of servant leadership. Employees’ sense of psychological empowerment gives them the feeling of competence and control in performing jobs. This inner confidence of empowerment is essential in realizing EVGB. Psychological empowerment, being at the heart of servant leadership characteristics and an antecedent of EVGB, is examined as an intervening variable through which servant leadership potentially exerts its influence towards EVGB. Furthermore, self-determination theory (SDT) stresses the vitality of autonomous motivation in shaping employee behaviors [26]. We operationalize autonomous motivation for the environment (AME) as being involved in those behaviors which align with one’s intrinsic goals and originates from the inner-self [27]. We relied on SDT [26] in proposing AME as another underlying mechanism between servant leadership and EVGB. AME is predicted to be essential in engaging an employee in voluntary green behavior. In doing so, we enhanced the explanatory power of the research model and better understanding of the path(s) through which servant leadership will lead to EVGB. The inclusion of AME, based on SDT, enabled us to establish a sequential mediation path that transmits the effect of servant leadership on EVGB through the underlying mechanism of psychological empowerment and AME.

Precisely, we aimed to offer four substantial contributions to the literature of servant leadership and EVGB. At first, despite a call to study the influence of various leadership styles on EVGB [10], as we understand, no empirical research has studied the direct impact of servant leadership on EVGB. Second, understanding the mechanism through which a leadership style exerts its influence towards employees’ outcomes is of vital importance for academicians and practitioners [28]. This research is an attempt, where psychological empowerment and AME are explored as independent and sequential mediators between the relationship of servant leadership and EVGB. Third, in line with the argument to develop a multilevel approach to enrich the understanding of environmental sustainability in organizational contexts [10,29], this research examined pragmatic predictors within a holistic model that included contextual (servant leadership) and personal level (psychological empowerment and AME) antecedents of EVGB. Finally, this research was conducted in Pakistan, a developing country where environmental laws are not up to standard. Particularly, Pakistan is among the top in the list of badly affected countries, due to environmental change and global warming [30]. Thus, the context of this research is highly meaningful for the practitioners.

We organized this article as follows: The coming Section 2 presents the hypotheses based on theoretical underpinnings and empirical evidences. Section 3 offers research methods encompassing the context, sampling and procedure, and measures adopted. Section 4 deals with the analysis, while in Section 5, discussion of the results is featured, along with theoretical and managerial implications. The last section, Section 6, includes conclusions, limitations, and future research avenues.

## 2. Theory and Hypotheses

### 2.1. Servant Leadership and Employees’ Voluntary Green Behavior

Employees’ voluntary green behavior refers to their discretionary actions that add to the sustainability of the environment and such behaviors are often not acknowledged by the organization’s formal reward system [31]. Such behaviors are neither specified in job descriptions nor systematically monitored and are usually not under the control of environmental management policies. EVGB helps an organization in developing its green strategies and may also be useful to shape the environmental caring behavior of citizens [5]. Typical instances of EVGB are saving paper at work, reducing energy use, helping colleagues to practice green behavior, and making recommendations for the protection of environment. Norton, Parker, Zacher and Ashkanasy [10] argued that the concept of EVGB is closely aligned with the notions of pro-environmental behaviors or organizational citizenship behavior for the environment (OCBE). It is at the discretion of employees to go beyond the limits of their job descriptions with respect to environment protection initiatives. This would be directly beneficial for organizational sustainability by preserving its resources and indirectly by conserving the natural environment. In line with this view, EVGB eventually helps in sustaining the health of Earth’s ecosystem.

The inception of servant leadership has its roots in Robert Greenleaf’s concept, who described the servant leader as a person with the aim to serve others and to ensure the fulfillment of others’ needs [32]. Such leaders think beyond their own interest and are innately motivated to serve others [33]. Under the servant leadership philosophy, a leader demonstrates an altruistic character for the benefit of followers and helps them to grow by providing opportunities for their material and emotional gains [34]. Liden et al. [35] described servant leadership with characteristics including “i-Emotional healing, ii-Empowering, iii-Helping subordinates grow and succeed, iv-Putting subordinates first, v-Creating value for the community, vi-Having conceptual skills, and vii-Behaving ethically”. By “empowering followers”, we mean enabling them and boosting their capacities so that they can take initiatives and feel free to act in their own way. Taking care of the community is an additional characteristic of servant leaders which distinguishes them from other types of leaders. Such leaders believe in grooming their followers on the same premises of paying attention and caring about the community in their routine activities. Due to their comprehensive vision about the organization and the surrounding environment, servant leaders act proactively to offer support, direction and resources to followers. SL has already shown its footprints on a wide range of organizational [36], group [37], and individual level outcomes [24,38]. Spears deemed servant leadership to be “a model that identifies serving others including employees, customers, and community as the number-one priority” [39]. Servant leaders, through their role-modeling of pro-environmental values, augment the followers’ positive perception of voluntary green behavior. Stewardship is a characteristic of servant leaders, wherein such leaders act as role models in performing social responsibilities. Tuan [40] employed environmental specific servant leadership (ESL) as a moderator between the relationship of corporate social responsibility (CSR) and OCBE, and found that servant leadership added synergy to CSR in predicting OCBE. In the same vein, Afsar et al. [41] investigated ESL as a moderator in the relationship of perceived CSR and pro-environmental behavior and found a significant interaction effect. Recently, servant leadership (environmentally specific), through the mediating effect of environmental engagement, displayed a positive influence on employees’ OCBE [42]. EVGB represents the ethical conviction of an employee and his/her commitment to reconcile the relationship between human society and nature while enhancing sustainability, aligning it with the central tenets of the philosophy of servant leadership, where serving others comprises nurturing employees as future servant leaders [32]. In line with this discussion, we postulate as follows:

**Hypothesis** **1.** *Servant leadership is positively related to EVGB*.

### 2.2. Autonomous Motivation for the Environment as a Mediator

The theme of self-determination theory (SDT) affirms that people can have different types as well as different levels of motivation [43]. According to SDT, there are three types of motivation that an individual can have, including amotivation, controlled motivation, and autonomous motivation [44]. Autonomous motivation confirms that individuals pursue those actions that are concordant and consistent with their underlying self [26]. Autonomous motivation accentuates that the self-determination of individuals includes identified motivation, integrated motivation and intrinsic motivation [26]. Under identified motivation, an individual performs those actions that are consistent with his/her goals and values. Within integrated motivation, “people have a full sense that the behavior is an integral part of who they are, that it emanates from their sense of self and is thus self-determined” [26]. The last component of autonomous motivation is the intrinsic motivation wherein people perform those actions that are inherently exciting or pleasing. Generally, people with autonomous motivation experience self-endorsement or volition of their actions [45]. Existing research has established the positive influence of AME with various types of pro-environmental behaviors, including resource conserving, recycling, etc. [46]

SL is built on the principle that leaders who place emphasis on compassion, moral behavior, and prioritize the needs of their followers are those who have the finest capabilities to motivate employees [32]. Despite theoretical support, empirical evidence lacks in establishing the relationship between servant leadership and autonomous motivation. However, a couple of studies highlight the positive influence of servant leadership on intrinsic motivation, a component of autonomous motivation [47,48]. SDT [43] argued that certain contextual and social factors, like leadership, may facilitate in shaping an individual’s autonomous motivation. Leadership can foster AME among employees by offering them assistance in internalizing green values [49]. Servant leaders’ support for the environment augments the sense of competence and autonomy of employees, and this provides the necessary ingredients for AME. Servant leaders inculcate self-sacrificing behavior in their employees for the greater good of society, e.g., protecting the environment. Thus, servant leadership is positively associated with AME. Autonomous motivation stimulates various workplace behavioral outcomes [26], including organizational citizenship behavior for the environment [12,49], and pro-environmental behavior [15,50,51]. Being purely intentional, AME is more likely to enhance EVGB because significance of the environment is consistent with their values and engaging in such activates gives them pleasure. Consequently, EVGB is believed to be more self-determined [52].

Further, the existing literature identified AME as a mediator in the relationship of supervisory support for the environment with OCBE [12], and transformational leadership for the environment with employees’ pro-environmental behavior [51]. Most recently, AME has also been investigated as a predictor of OCBE and as an intervening variable between responsible leadership and the OCBE relationship [49]. The primary consideration of employees in engaging in voluntary green behaviors is the satisfaction of their inner self and not financial gain. The activities of the employees pertaining to environment protection, elicited by autonomous motivations, are aligned with their values, goals and interests [26,53]. While working with servant leaders, employees’ environmental friendly belief enhances their AME, which in turn steers them to act in a way that is harmonious with that belief [54], such as in the form of EVGB. Employees may stimulate their AME by internalizing the values and environmental goals of their servant leadership, which results in the enhancement of their active participation in voluntary green behaviors. Therefore, we hypothesize:

**Hypothesis** **2a.** *Servant leadership is positively related to autonomous motivation for the environment*.

**Hypothesis** **2b.** *Autonomous motivation for the environment is positively related to EVGB*.

**Hypothesis** **2c.** *Autonomous motivation for the environment mediates the relationship between servant leadership and EVGB*.

### 2.3. Psychological Empowerment as a Mediator

Psychological empowerment theory maintains that when employees feel empowered, they are likely to take proactive initiatives toward their work and deliver beyond their mandate [55]. Psychological empowerment is a motivational variable comprised of four dimensions: i-meaning, ii-competence, iii-self-determination, and iv-impact [56]. Meaning gives employees an impression of the importance and value of their jobs [56]. Competence refers to employees’ self-belief or confidence in their abilities and skills, which are required to accomplish their assignments in the workplace. Furthermore, when employees feel that they have choice or freedom in the decision-making relevant to their jobs, this enhances their perception of self-determination [56]. Lastly, the impact reflects the degree to which employees believe that their jobs can make a difference in realizing the outcomes or objectives of the organization [56]. Realizing the importance of preserving the environment when accompanied by personal discretion over action is likely to lead employees towards voluntary green behavior. When employees start feeling proficient in accomplishing positive outcomes, their perception of their competence and impact also increases [57]. Psychological empowerment is likely to be associated with extra-role behaviors like EVGB, where the performance of such behaviors is not essentially required and employees’ consider such behaviors as valuable for the protection of the environment. The existing literature supports our argument that employees’ psychological empowerment has shown a positive association with OCBE [25], a construct synonymous with EVGB.

Nonetheless, scholars claimed that psychological empowerment would be one of the underlying mechanism of servant leadership to exert its impact on followers’ outcomes [35,58], and there is a paucity of empirical evidence to verify this argument [59]. Servant leaders are expected to nurture the psychological empowerment of their subordinates by focusing on their needs satisfaction, offering autonomy in their domains, equitable treatment, accountability, and transparency in dealings. Servant leaders influence the employees’ outcomes through needs satisfaction and by offering personal development opportunities [35,60], fair treatment and respect, instead of considering them as a source of personal or organizational advantage [59]. Such leaders realize their followers about the value of their jobs in a broader picture [59]. Previously, scholars have investigated the impact of servant leadership on employees’ psychological empowerment and resulting positive outcomes [59,61]. In addition, servant leaders inculcate a sense of competence in their followers by giving them chances to acquire new skills through openness to experience and training. Besides, servant leadership believes in participative decision making [62], which possibly heightens the self-determination perception of followers [33,62]. Lastly, servant leadership realizes their followers’ about their jobs’ impact on the overall organization and society, which portray the importance of their jobs. To conclude, servant leaders boost the psychological empowerment of their followers by way of positive perception of meaning, competence, self-determination, and impact.

Seibert et al. [63], in their meta-analysis, established that general organizational citizenship behavior of employees was a behavioral consequence of psychological empowerment. They further synthesized the literature and presented an integrated model of the predictors and consequences of psychological empowerment, wherein contextual antecedents (like leadership) are followed by behavioral outcomes (like OCBE). We utilized that model for the directional guidance of hypothesized relationships. In the extant empirical literature of servant leadership, the mediating role of employees’ psychological empowerment was examined to predict employee engagement [64], employee commitment and job satisfaction [65], and employees’ innovative work behavior [61]. Besides, and not only in relation to servant leadership, employees’ psychological empowerment has also been introduced as a mediator in the relationships of other leadership styles (transformational, ethical, participative) with various employees’ related outcomes [66]. Psychological empowerment has also shown positive findings as a mediator in the relationship of servant leadership with employees’ general OCB [67]. In the recent past, Lamm, Tosti-Kharas and King [25] found promising results about psychological empowerment as a mediator between the relationship of perceived organizational support for the environment and employees’ OCBE. This discussion leads us to formulate:

**Hypothesis** **3a.** *Servant leadership is positively related to psychological empowerment*.

**Hypothesis** **3b.** *Psychological empowerment is positively related to EVGB*.

**Hypothesis** **3c.** *Psychological empowerment mediates the relationship between servant leadership and EVGB*.

### 2.4. Psychological Empowerment and Autonomous Motivation for the Environment

The four cognitions of psychological empowerment (i-meaning, ii-competence, iii-self-determination, and iv-impact) align closely with the three psychological needs (relatedness, competence, and autonomy) of SDT. These three psychological needs serve as the prerequisites for autonomous motivation. When employees have a sense of connectivity with peers and share mutual goals, this serves as the basis of their need for relatedness [45]. The need for competence is realized through the positive perception of employees’ regarding their capability of accomplishing a given task. Lastly, when employees perceive that they have influence and control over their actions and the subsequent consequences, this satisfies their need for autonomy. Thus, we view the association between psychological empowerment and autonomous motivation for the environment through the theoretical lens of SDT. An autonomy driven organizational culture offers information to the employees in a non-controlling way, provides a choice of decisions, and facilitates self-initiation, thereby lifting their level of autonomous motivation. Without controlled motivation, to engage employees in challenging extra-role behaviors like EVGB, psychological empowerment ensures the necessary impetus for their autonomous motivation [26]. Employees’ feeling of being psychologically empowered allows them to internalize the values associated with engagement in self-determined tasks. As a result, there is an increased likelihood of employees engaging in voluntary green behaviors, not because they are rewarded or compelled by social pressures, but due to the fact that they recognize such behaviors as an essential part of their sense of self, and this is therefore self-determined [26]. Hence, psychological empowerment is required to maintain and keep alive the level of employees’ autonomous motivation. To strength test it with empirical evidence, Masood and Afsar [68] found that psychological empowerment had a positive influence on the employees’ intrinsic motivation, a component of autonomous motivation. Therefore, we hypothesize:

**Hypothesis** **4.** *Psychological empowerment is positively related to autonomous motivation for the environment*.

### 2.5. Sequential Mediation of Psychological Empowerment and AME

**Figure 1:**
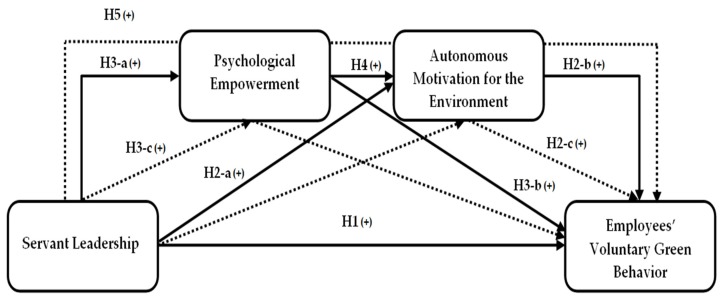
Hypothesized Model. Note: Plain lines represent the direct relationships, while dotted lines show the mediation relationships.

The existing scholarship has established the positive influence of servant leadership on employees’ psychological empowerment [61], intrinsic motivation [48], and employees’ extra-role behaviors [42]. In the preceding sections of this article, it has been further accentuated that psychological empowerment and autonomous motivation for the environment would be the mechanisms through which servant leadership exerts its influence in predicting EVGB. Additionally, psychological empowerment were also found to be closely associated with autonomous motivation. By integrating these arguments, this discussion can be led to a next logical level, where it is anticipated that when servant leadership enhances the psychological empowerment of employees, they feel more autonomous motivated towards the environment, which eventually leads to their enhanced voluntary green behavior. The rationale to postulate sequential mediation is fully supported by the concepts of servant leadership, psychological empowerment and self-determination theories. So, we postulate:

**Hypothesis** **5.** *Psychological empowerment and AME sequentially mediate the relationship between servant leadership and EVGB (Figure 1)*.

## 3. Methods

### 3.1. Research Context

The selection of respondents from the power sector organizations of Pakistan is significant because this sector is the largest source of greenhouse gas emission in Pakistan, accounting for almost 50% [69]. Besides, this sector is the second largest employer, having its employees all across the country.

### 3.2. Sample and Procedure

The HR department of the Pakistan Electric Power Company (administrative authority in that sector) requested to provide a list of employees and their immediate supervisors. They shared a list of 4902 employees in total, all working in non-managerial positions, and their immediate supervisors. The list contained names, designations, and email addresses of the potential respondents. Two separate questionnaires were prepared with the help of Google Docs, one each for employees and their immediate supervisor. The questionnaire for subordinates contained questions related to servant leadership, psychological empowerment and AME, while the questionnaire for supervisors consisted of questions related to EVGB. Data were collected in the month of September 2019. Systematic sampling was employed to select the participants of this research. Web links of the questionnaires were sent to respondents through an email requesting their voluntary participation. The questionnaires were administered in English, as it is the official language of Pakistan. Systematically, every 7th employee and his/her immediate supervisor were shortlisted as potential respondents for this research. A total of 701 questionnaires were administered to subordinates. To prepare dyadic responses, 264 immediate supervisors of 701 employees were requested to participate in the survey. A total of 329 questionnaires were received from the subordinates, and 337 questionnaires were received from supervisors. Finally, the matched responses included a dyad of 315 employees and their immediate supervisors with a response rate of 44.94 percent. Table 1 demonstrates the demographics of the respondents.

### 3.3. Measures

All the constructs were evaluated on a seven-point Likert scale ranging from ‘1’ = strongly disagree to ‘7’ = strongly agree.

Employees’ voluntary green behavior was measured by a ten-item scale for organizational environmental citizenship behavior, developed by Robertson and Barling [70]. Supervisors evaluated the voluntary green behavior of their immediate subordinates. A sample item includes “At work, he/she recycles whenever possible”. The scale has a 0.97 Cronbach’s alpha value. Employees assessed the servant leadership style of their immediate supervisors on a 7-item measure, advanced by Liden et al. [71] to assess global servant leadership. Sample items included “My leader emphasizes the importance of giving back to the community”. Cronbach’s alpha of the measure was above 0.8.

Autonomous motivation for the environment was evaluated by the ratings of employees on a twelve-item scale from the Motivation towards Environment Scale study of Pelletier et al. [72] Based on the principles of self-determination theory, we considered AME as a variable consisting of identified motivation, integrated motivation, and intrinsic motivation. Every question preceded “Why are you doing things for the environment?” One of the items included “it is pleasure in improving quality of environment” with a Cronbach’s alpha value of 0.91.

Psychological empowerment was assessed through the employees’ rating on a twelve-item scale developed and validated by Spreitzer [56]. This measure has four sub-dimensions, each of which has three items. One of the items was “I have considerable opportunity for independence and freedom in how I do my job”. The Cronbach’s alpha of all the four dimensions was more than 0.75.

### 3.4. Control Variables

Abrahamse and Steg [73] suggested that the demographic variables of the respondents can influence their green behaviors. We took respondents’ gender, age, education, and experience as control variables.

## 4. Results and Analysis

The analysis was carried out by employing variance-based SEM, using partial least squares [74,75] through the Smart-PLS 3.2.8 (Boenningstedt, Germany) software [76]. In recent years, partial least squares structural equation modeling (PLS-SEM) has shown its footprints over diversified disciplines including marketing, accounting, human resource management, and many others [77]. Rather than following the crowd, authors have preferred PSL-SEM for several reasons. At first, PLS-SEM allows for the analysis of complex models with multiple constructs, indicators, and relationships [78]. Second, the latest guidelines on the use of PLS-SEM proved its superiority over other techniques in the assessment of mediation analysis [79]. Third, psychological empowerment and AME were designed as second-order constructs, and PSL-SEM is a better choice for dealing with the models that have higher order constructs [78,79]. Fourth, PLS-SEM offers better ‘statistical power’ [80]. Lastly, it is considered to be equally efficient for exploratory- and prediction-oriented research [81]. A two-stage approach, namely involving (i) measurement model evaluation, and (ii) structural model evaluation, was employed to analyze the results of PLS-SEM [82].

### 4.1. Measurement Model Evaluation

In this section, the distinction between ‘reflective’ and ‘formatively’ designed constructs needed to be considered at the outset. All the constructs designed are ‘reflective’ in nature. Besides, psychological empowerment and AME were designed as second-order reflective-reflective constructs. We followed the two-step approach for the measurement model evaluation of second-order constructs. The latent variable scores of the lower order reflective constructs were used as manifest variables of the higher order constructs.

#### 4.1.1. Reliability

At first, the individual indicator’s reliability was ensured by standardized factor loadings and it was established when an indicator had a standardized factor loading of ≥0.70 on its associated construct [83]. The second step requires examining the internal consistency-reliability of the constructs. The latest guidelines on reporting the results of PLS-SEM recommended the use of Dijkstra and Henseler’s [84] ‘*ρ*_A_’ as an approximately precise measure of reliability over traditional Cronbach’s alpha and composite reliability. Table 2 presets values of factor loadings, *ρ*_A_, Cronbach’s alpha, and ‘CR’ that are between 0.7 to 0.9 [81], and unanimously confirms internal consistency/reliability of the first and the second order reflective constructs.

#### 4.1.2. Convergent Validity

The 3rd stage of the measurement model evaluation requires the establishment of the convergent validity of the constructs, and for this purpose, average variance extracted (AVE) is a widely used metric [83]. An AVE value of 0.5 or greater means that the construct explains more than half of the variance of the indicators which constitute that construct [85]. The findings presented in Table 2 confirm that the AVE values of all the constructs are more than the recommended value of the threshold.

#### 4.1.3. Discriminant Validity

The latest guidelines on the evaluation of the PLS-SEM measurement model advocate the use of the heterotrait–monotrait (HTMT) ratio of correlations, over the traditional Fornell and Larkers approach [74], to establish the discriminant validity of the constructs. When the constructs have a higher conceptual distinction, a HTMT threshold of up to 0.85 was recommended [83]. The findings in Table 3 present the mean and standard deviation, along with ensuring the discriminant validity.

### 4.2. Structural Model Evaluation

Relationships among the dependent and independent variables were assessed, while analyzing the structural model through the size and direction of path coefficients, values of the coefficient of determination, and *t*-values [77,86]. Following recent studies, we treated psychological empowerment and AME as higher order (single factor) constructs [12,63,66]. Before we proceeded further, assessment of the collinearity was carried out with the variance inflation factor (VIF) technique and all the values accorded below the threshold of 3 [81], stressing that the model was not contaminated due to common method bias [87].

#### 4.2.1. Path Coefficient (β)

A path weighting scheme was run with default settings of the Smart-PLS 3.2.8 software. The bootstrapping procedure was run with 5000 subsamples, and no sign change option was chosen. Significance levels for one-tailed testing, along with percentile bootstrap were the other options selected to run the procedure. For the statistical significance of hypotheses, the value of path coefficients should be established through the percentile bootstrap confidence interval [88] and its direction should be consistent with the respective hypotheses. Table 4 represents the detail of the hypotheses, where the findings are confirmed through *t*-values and the percentile bootstrap confidence interval. We observed 1.96 as the cutoff criterion for t-statistics [66,89]. The results reveal that all hypothesized relationships are significantly supported, except H1. Besides, from the control variables, the influence of Education on EVGB is significant, while other control variables are not significant.

#### 4.2.2. Coefficient of Determination (R^2^)

The R^2^ value of the endogenous construct represents the within-sample predictive power of the structural model [78]. As a rough estimate, the R^2^ value of 0.25, 0.50, and 075 represents the ‘weak’, ‘moderate’, and ‘strong’ traits, respectively [78]. The R^2^ value for the key target construct of employees’ voluntary green behavior is 72.2%, which means that all the antecedents explained had substantial variance in EVGB. The R^2^ values of all the endogenous constructs are listed in Table 4.

#### 4.2.3. Blindfolding (Q^2^)

PLS-SEM contains a supplementary method to evaluate the predictive ability of the structure through blindfolding approach denoted by Q^2^ [81,90]. To find the values of Q^2^ in Smart-PLS 3.2.8, a blindfolding process using a cross-validated redundancy approach was employed. More than zero values for all the endogenous constructs were obtained, which ensures the predictive accuracy of the model [81,90]. Table 4 presents the Q^2^ values of the endogenous constructs.

#### 4.2.4. Out-of-Sample Predictive Quality-PLSpredict

Lastly, the out-of-sample predictive quality of the model was assessed through the Smart-PLS option of PLS predict, by following the procedure proposed by Shmueli [91]. The Q^2^ predict values for all the indicators of the key target construct of employees’ voluntary green behavior were found to be positive (see Table 5). Moreover, for all the indicators of EVGB, the PLS-SEM results have a smaller prediction error compared to the linear model benchmark. Therefore, the model established a high predictive power [92].

### 4.3. Mediation Analysis

**Figure 2:**
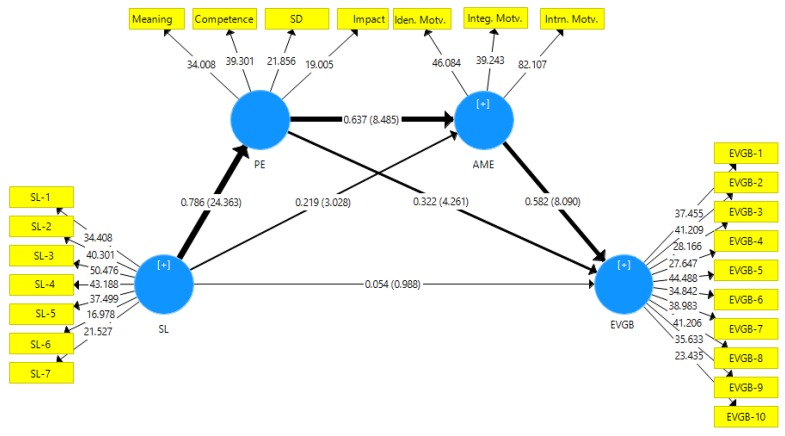
Structural model.

To establish mediation, the significance of the direct, indirect effect and total effect needs to be assessed [93,94]. In this research, direct effect is the value of path coefficient (*β*) from servant leadership to EVGB. Indirect effect is the product of the direct effect from servant leadership to mediating variable (psychological empowerment and/or AME) and the direct effect from those mediating variable(s) to EVGB. Then, the total effect is the sum of the direct effect and indirect effect. To obtain results, 5000 subsamples were bootstrapped with 315 observations per subsample. No sign change option to determine the significance of the path coefficients with *p* less than 0.05 two-tailed was the other setting. The significance of the hypotheses was established through a confidence interval, such that it should not contain a ‘0’ value [78]. The results of the mediation analysis are shown in Table 6 and Figure 2.

## 5. Discussion and Implications

### 5.1. Discussion

This research intended to examine the impact of servant leadership on employees’ voluntary green behavior, first directly and then through independent and sequential mediation of psychological empowerment, and autonomous motivation for the environment. Findings revealed that the direct impact of servant leadership on EVGB was not supported. This result is inconsistent with the literature, where the direct or indirect effect of servant leadership was investigated on employees’ organizational citizenship behavior for the environment [40,41,42], a construct similar to EVGB. The reason behind this finding is that both psychological empowerment and autonomous motivation for the environment fully mediates the influence of servant leadership on EVGB. It is pertinent to state that without introducing both the mediators, i.e., psychological empower and AME, servant leadership has a positive significant effect on EVGB. However, after introducing multiple mediations, the effect of servant leadership on EVGB becomes insignificant.

The finding that servant leadership has a positive influence on autonomous motivation for the environment is congruent with the existing literature, where servant leadership has shown its positive impact on intrinsic motivation [48], a component of autonomous motivation. The more a leader demonstrates the servant leadership style, the more the followers feel autonomously motivated. Then, the positive impact of AME on EVGB can be explained through the lens of SDT, which asserts that employees’ voluntary behaviors can be channelized through their enhanced autonomous motivation. This finding is in accordance with the recent studies, where the autonomous motivation for the environment leads to employees’ extra role behavior towards the environment [12,17]. Furthermore, the results confirm the mediating role of autonomous motivation for the environment between servant leadership and the EVGB relationship. Servant leaders consider the benefit of all the stakeholders, within and outside the organization, and their support for the environment provides the necessary nutrition of AME, which channelizes EVGB. This means that AME is one of the mechanisms that servant leadership opts to enhance; EVGB. This finding is partially consistent with the existing empirical research, where AME served the intervening role in predicting employees’ extra role behavior towards the environment [12,49,51], and this strongly corroborated with the application of self-determination theory.

Next, the servant leadership is hypothesized to have a positive impact on employees’ psychological empowerment. Results strongly supported this postulation and validated the earlier research, where servant leadership was found to be a significant predictor of employees’ psychological empowerment [64]. The empowerment of followers is considered as one of the vital characteristics of servant leaders. Furthermore, this study supported the positive influence of psychological empowerment on EVGB. The dimensions of psychological empowerment, including meaning, competence, self-determination and impact, enable employees to take initiatives in performing extra-role behaviors like EVGB. Furthermore, the hypothesis regarding the mediating role of employees’ psychological empowerment between the relationship of servant leadership and EVGB has also been supported. This result is partially consistent with the earlier studies, where psychological empowerment was investigated as the moderator between servant leadership and employees’ outcomes [61] other than EVGB.

The introduction of multiple mediators sequentially offers a deeper insight into the influencing mechanism of servant leadership towards EVGB. The findings showed that psychological empowerment and AME sequentially mediate the relationship between servant leadership and EVGB. This is a unique empirical contribution of this research and draws its support by integrating self-determination and psychological empowerment theories. Interestingly, after the introduction of multiple mediators in the structural model, the impact of servant leadership on EVGB becomes non-significant, which proves that psychological empowerment and AME fully mediate the impact of the servant leadership on EVGB. Alternately, whatever the positive influence servant leadership exerted on EVGB was through the mediating mechanisms of psychological empowerment and AME. As far as the relative importance of psychological empowerment and autonomous motivation for the environment is concerned, psychological empowerment offers more significant intervening mechanisms. We explored another interesting finding while analyzing sequential mediation of psychological empowerment and AME. The path from servant leadership to AME remains marginally significant, which means that servant leadership largely influences AME through the intervening role of psychological empowerment. Thus, this study highlights the critical value of employees’ psychological empowerment to servant leaders, while shaping AME and EVGB.

### 5.2. Theoretical Implications

This research contributes in various ways to the literature of servant leadership and employees’ voluntary green behavior. At first, Norton, Parker, Zacher and Ashkanasy [10] invited researchers to explore the influence of different leadership styles on EVGB, and there is a paucity of research that has examined the influence of servant leadership style on employees’ voluntary green behavior. Prior research has investigated only the moderating role of servant leadership in the relationship of CSR and OCBE [40]. Therefore, this research is an attempt to advance the knowledge stream of servant leadership by investigating its direct relationship with EVGB. When it comes to protecting the environment, servant leaders are considered to care for the environment over their own or the organization’s financial gain and promote pro-environmental values among the stakeholders, including employees [40,42].

Secondly, this research enriches the literature on the influencing mechanism of servant leadership in predicting employees’ outcomes. Understanding the influencing mechanism of servant leadership has already been desired by the researchers [28]. We proposed psychological empowerment and AME as the underlying mechanisms through which servant leadership would enhance EVGB. Besides, we offered a sequential mediation mechanism where servant leadership transmits its influence in the form of employees’ psychological empowerment, which then lifts their autonomous motivation for the environment, and finally, they demonstrate higher levels of voluntary green behaviors. These mechanisms are fully backed by the concepts of self-determination theory (SDT) and are highly valuable for the research fraternity, due to their comprehensive depiction of the underlying working of servant leadership. Furthermore, we advanced the literature on SDT by establishing the influence of servant leadership with autonomous motivation for the environment. None of the existing research has examined this relationship empirically.

Third, we advanced the knowledge of EVGB by investigating its antecedents at a contextual and personal level in a single conceptual model. Although limited empirical studies have already investigated psychological empowerment and AME as independent antecedents of EVGB, conceptualization of these antecedents in the form of sequential mediation is a unique theoretical insight for EVGB literature. This research specifically enhances the nomological network of EVGB, as well as servant leadership constructs.

### 5.3. Practical Implications

We offered organizational practitioners a roadmap to ‘grow’ their employees into ‘environmental activists’ for the sustainable development of their organization. First, this research reinforced the importance of servant leadership. Findings revealed that servant leadership positively influences employees’ psychological empowerment and AME, that in turn leads to their enhanced voluntary green behavior. The top management of organizations, while selecting managers, should give priority to those individuals who can demonstrate servant leadership in organizations. Besides, organizations may offer its managers training and development opportunities that can enhance their level of servant leadership. The concept of the servant leader is based on the belief in creating value for the community, serving others, delegating power, and empowering followers to grow and succeed in their professional and personal lives. Such leaders, through regular interaction with subordinates, teach them the importance of giving back to the community and enhance EVGB. It can lift a manager’s level, as being a servant leader improves interaction with employees, and this can enhance EVGB. Secondly, the importance of employees’ psychological empowerment for their autonomous motivation for the environment and engagement in voluntary green behavior in the workplace has also been highlighted. Organizational managers should realize their subordinates’ about their capabilities, competencies and should afford them freedom in doing their jobs. Third, organizations should have a keen focus on the employees’ autonomous motivation for the environment and continuously take initiatives that can enhance or at least keep this motivation alive.

## 6. Limitations, Future Research Directions, and Conclusions

Despite numerous theoretical and practical offerings, this research is not without limitations. First, the cross-sectional nature of the data confines this research’s ability to establish any inference about causality. We suggest longitudinal investigations of this study’s model to overcome this limitation. Second, the data for this research were collected from employees and their immediate supervisors employed in the power sector organizations of Pakistan. Though the context of this research is highly valuable for practitioners, it is different from the context of other sector/industries and countries. Thus, the findings of this research need care while generalizing. For enhanced generalizability, similar studies in different sectors/industries and countries are proposed. Third, we employed general servant leadership in our conceptual model, due to the non-availability of a valid scale to measure environment specific servant leadership. We invite scholars to prepare and validate an environment specific servant leadership scale, so that future researchers can employ environment specific servant leadership in their research models. Fourth, this research included psychological empowerment and AME as mediators; aspirant researchers recommend the examination of other factors at the organizational, team, and individual level as mediating and/or moderating variables. Fifth, we gathered data from a single source and employed the same technique, which may have caused some degree of bias. Lastly, we recommend that aspirant researchers model AME and psychological empowerment as lower order constructs, dimensions level, in their theoretical models.

In the burgeoning research area of employees’ green behavior at the workplace, we tried to identify the influencing mechanisms of servant leadership towards employees’ voluntary green behavior. A multi-theory perspective comprising self-determination and psychological empowerment theories could be employed to develop a multi-level conceptual model of this research, including servant leadership, psychological empowerment, and AME as antecedents of EVGB. Servant leadership was found to be a substantial predictor of psychological empowerment, autonomous motivation for the environment, and VEGB. In addition, psychological empowerment and AME was validated as being a predictor of EVGB.

## Figures and Tables

**Table 1:** Demographics of the respondents.

Category	Total Respondents		Matched Responses		
Supervisors	143		315		
Employees	315		315		
	**Gender**	**Male**		**Female**	
Supervisors		124 (87%)		19 (13%)	
Employees		233 (74%)		82 (26%)	
	**Age (in years)**	**18–25**	**26–35**	**36–45**	**Above 45**
Supervisors		30 (21%)	43 (30%)	53 (37%)	17 (12%)
Employees		79 (25%)	132 (42%)	82 (26%)	22 (07%)
	**Experience (in years)**	**01–05**	**06–10**	**11–15**	**Above 15**
Supervisors		24 (17%)	43 (30%)	59 (41%)	17 (12%)
Employees		57 (18%)	88 (28%)	135 (43%)	35 (11%)
	**Education**	**Below Graduate**	**Graduated**	**Master**	**Above Master**
Supervisors		19 (13%)	32 (22%)	80 (56%)	13 (09%)
Employees		66 (21%)	151 (48%)	82 (26%)	16 (05%)
	**Respondents’ Level**				
Supervisors	Managers 33 (23%)	Deputy Managers 44 (31%)		Assistant Managers 63 (44%)	
Employees	Support Staff/Office Staff 315 (100%)				

**Table 2:** Results of the measurement model.

Construct	Items	Loadings	C (*α*)	rho-A	CR	AVE	VIF
**Step I:** Assessment of the Measurement Model for First-Order Constructs							
Employees’ Voluntary Green Behavior	VGB1	0.845	0.920	0.926	0.935	0.728	2.68
	VGB2	0.860					
	VGB3	0.876					
	VGB4	0.846					
	VGB5	0.839					
	VGB6	0.881					
	VGB7	0.859					
	VGB8	0.870					
	VGB9	0.862					
	VGB10	0.844					
Servant Leadership	SL1	0.823	0.923	0.928	0.939	0.687	2.57
	SL2	0.850					
	SL3	0.879					
	SL4	0.879					
	SL5	0.856					
	SL6	0.737					
	SL7	0.767					
Meaning	ME1	0.803	0.907	0.918	0.931	0.744	2.34
	ME2	0.823					
	ME3	0.809					
Competence	CO1	0.875	0.825	0.839	0.855	0.740	2.29
	CO2	0.862					
	CO3	0.843					
Self-Determination	SD1	0.833	0.802	0.825	0.850	0.760	2.63
	SD2	0.729					
	SD3	0.778					
Impact	IM1	0.814	0.826	0.837	0.849	0.772	2.08
	IM2	0.740					
	IM3	0.847					
Intrinsic Motivation	Intrn. Motv.1	0.893	0.831	0.835	0.851	0.754	1.93
	Intrn. Motv.2	0.806					
	Intrn. Motv.3	0.739					
	Intrn. Motv.4	0.802					
Integrated Motivation	Integ. Motv.1	0.757	0.863	0.879	0.886	0.719	1.87
	Integ. Motv.2	0.781					
	Integ. Motv.3	0.821					
	Integ. Motv.4	0.807					
Identified Motivation	Iden. Motv.1	0.817	0.803	0.814	0.845	0.733	1.95
	Iden. Motv.2	0.797					
	Iden. Motv.t3	0.804					
	Iden. Motv.4	0.862					
**Step-II:** Assessment of the Measurement Model for Second-Order Constructs							
Psychological Empowerment	Meaning	0.813	0.778	0.792	0.803	0.698	2.61
	Competence	0.851					
	Self-Determination (SD)	0.799					
	Impact	0.820					
Autonomous Motivation for the Environment	Intrinsic Motivation	0.819	0.789	0.798	0.811	0.705	2.20
	Integrated Motivation	0.793					
	Identified Motivation	0.810					

**Note:** rho A = Dijkstra and Henseler’s ρA; CR = Composite reliability; AVE = Average variance extracted; VIF = Variance Inflation Factor. Results obtained through SmartPLS algorithm function.

**Table 3:** Standard deviation and discriminant validity through HTMT approach.

	Mean	SD	EVGB	PE	AME	SL
Employees’ Voluntary Green Behavior	3.09	1.14				
Psychological Empowerment	3.17	0.93	0.825			
Autonomous Motivation for the Environment	3.12	1.06	0.803	0.811		
Servant Leadership	2.96	1.05	0.712	0.807	0.829	

**Table 4:** Results of the structural model.

Structural Paths		Direct Effect	*t*-Value	95% Percentile Confidence Interval	Decision
**Control variables path**					
	Gender -> EVGB	0.063	01.170	[−0.012, 0.187]	* n.s
	Age -> EVGB	0.112	01.244	[−0.025, 0.213]	* n.s
	Education -> EVGB	0.230	02.095	[0.241, 0.332]	**Significant**
	Experience -> EVGB	0.097	01.119	[−0.031, 0.245]	* n.s
**Direct hypothesized path**					
**H1:**	SL -> EVGB	0.054	01.004	[−0.032, 0.144]	**Not-Supported**
**H2-a:**	SL -> AME	0.220	03.046	[0.115, 0.351]	Supported
**H2-b:**	AME -> EVGB	0.582	08.420	[0.465, 0.694]	Supported
**H3-a:**	SL -> PE	0.786	25.785	[0.736, 0.832]	Supported
**H3-b:**	PE -> EVGB	0.322	04.425	[0.199, 0.446]	Supported
**H4:**	PE -> AME	0.637	08.309	[0.498, 0.750]	Supported
**Quality indicators of the structural model**					
**R^2^_Employees’ Voluntary Green Behavior_ = 0.722**			**Q^2^_Employees’ Voluntary Green Behavior_ = 0.452**		
**R^2^_Autonomous Motivation for the Environment_ = 0.574**			**Q^2^_Autonomous Motivation for the Environment_ = 0.325**		
**R^2^_Psychological Empowerment_ = 0.426**			**Q^2^_Psychological Empowerment_ = 0.279**		

**Note:***p* < 0.01, *n.s = Not significant.

**Table 5:** Results of PLSpredict.

Indicator	Q^2^ Predict	PLS-SEM		Liner-Model Benchmark	
		RMSE	MAE	RMSE	MAE
EVGB-1	0.399	**1.367**	**1.042**	1.417	1.071
EVGB-2	0.343	**1.429**	**1.076**	1.475	1.110
EVGB-3	0.399	**1.349**	**1.010**	1.364	1.019
EVGB-4	0.309	**1.431**	**1.103**	1.453	1.118
EVGB-5	0.354	**1.386**	**1.018**	1.396	1.025
EVGB-6	0.359	**1.401**	**1.065**	1.432	1.074
EVGB-7	0.336	**1.412**	**1.062**	1.450	1.099
EVGB-8	0.395	**1.375**	**1.069**	1.409	1.093
EVGB-9	0.387	**1.365**	**1.007**	1.376	1.012
EVGB-10	0.420	**1.282**	**0.907**	1.301	0.963

**Note:** RMSE (Root mean squared error), MAE (Mean absolute error). After comparison of PLS-SEM results with Liner-model benchmark, the bold values represent where the prediction error is lesser.

**Table 6:** Results of the mediation analysis.

Hypothesized Path	Direct Effect	Indirect Effect	Total Effect	95% Percentile CI	Decision
**H2-c:** SL -> PE -> EVGB	0.182	0.545	0.727	[0.468, 0.624]	Supported
**H3-c:** SL -> AME -> EVGB	0.190	0.536	0.726	[0.464, 0.609]	Supported
**H5:** SL -> PE -> AME -> EVGB	0.191	0.474	0.665	[0.396, 0.547]	Supported

**Note:** CI: Confidence Interval.
